# The epidemiology of boys’ youth lacrosse injuries in the 2015 season

**DOI:** 10.1186/s40621-016-0068-5

**Published:** 2016-02-01

**Authors:** Zachary Y. Kerr, Shane V. Caswell, Andrew E. Lincoln, Aristarque Djoko, Thomas P. Dompier

**Affiliations:** 1 Datalys Center for Sports Injury Research and Prevention, 401 W. Michigan St., Suite 500, Indianapolis, IN 46202 USA; 2 George Mason University, Sports Medicine Assessment, Research & Testing (SMART) Laboratory, 10900 University Blvd. MS 4E5, Manassas, VA 20110 USA; 3 MedStar Sports Medicine Research Center, 201 E. University Parkway, 764 Bauernschmidt Bldg, Baltimore, MD 21218 USA

**Keywords:** Epidemiology, Youth sports

## Abstract

**Background:**

Participation in boys’ youth lacrosse has dramatically increased in recent years. Yet, research on the incidence of youth lacrosse injuries is limited. This study describes the epidemiology of boys’ youth lacrosse injuries.

**Findings:**

Aggregate injury and exposure data was collected from 550 boys’ youth lacrosse players (aged 9–15 years) from eight leagues in four states. Injury frequencies and rates with 95 % confidence intervals (CI) were calculated. Rate ratios (RR) accounting for clustering within league compared game and practice injury rates. During the 2015 season, 155 injuries were reported for a rate of 12.98/1000AE (95 % CI:10.93-15.02). Most injuries occurred during games (60.0 %), resulted in time loss <24 h (83.9 %), and were in the U13/U15 divisions (69.0 %). Most injuries were to the lower extremity (45.2 %), and diagnosed as contusions (51.6 %). Ten concussions (6.5 %) were reported, with seven occurring in the U13/U15 divisions. All injuries resulting in time loss ≥24 h in the U9/U11 divisions were concussions. Most injuries were due to equipment contact, particularly stick contact (35.5 %) and ball contact (14.2 %). Injury rates were higher in games than practices overall (RR = 2.90; 95 % CI:1.81-4.89), and for concussions only (RR = 4.51; 95 % CI:1.89-11.03). Between the U9/U11 and U13/U15 divisions, the overall-injury rate was higher in U9/U11 (RR = 1.23; 95 % CI:1.05-1.44).

**Conclusions:**

Our boys’ youth lacrosse injury rate was higher than those previously reported, but may be more precise given the larger sample. The large proportion of equipment contact injuries demonstrate the need to adopt currently available coaching instruction and age-appropriate US Lacrosse rules that could better protect youth players.

## Findings

### Introduction

Participation in youth lacrosse (≤15 years) has increased in recent years, with 2014 estimates of 279,771 youth participants in the United States (US Lacrosse “Participation…” 2015) Although lacrosse injury data are available at the high school and college levels (Dick et al. 2007; Xiang et al. 2014), youth level data are limited. The recent study involving male players aged 9–15 years estimated an injury rate of 8.7/1000 athlete-exposures (AE) from 22 injuries (Lincoln et al. 2014) Data utilizing larger samples of youth lacrosse players will aid the development of sports injury prevention strategies to reduce injury incidence and severity. This study describes the epidemiology of injuries in boys’ youth lacrosse in the 2015 season.

## Methods

This study employed a one-season observational cohort design. A total of 550 boys’ youth lacrosse players from eight leagues in four states (Indiana, Massachusetts, South Carolina, and Virginia) and between the ages of 9–15 (mean age: 12 ± 2) were followed over the 2015 season. The study protocol was approved by the Western Institutional Review Board (Puyallup, WA).

Data collection for youth lacrosse parallels that of previous youth football studies that have been explained in detail previously (Kerr et al. 2015). On-site athletic trainers (ATs) reported injury and exposure data from all games and practices into a single injury documentation application called the Injury Surveillance Tool (IST [Datalys Center, Indianapolis, IN]). All ATs received standardized training in the use of the IST. De-identified injury and exposure information were exported to a central database and reviewed by quality control staff on a weekly basis.

An injury was defined as an injury/illness occurring during a league-sanctioned game or practice that required AT evaluation (Kerr et al. 2015). A time loss (TL) injury restricted participation for ≥24 h; a non-time loss (NTL) injury restricted participation for <24 h. An athlete-exposure (AE) was defined as one player participating in one game or one practice. Data were analyzed using SAS-Enterprise Guide software (version 5.1; SAS Institute Inc., Cary, NC). Frequencies, injury rates, and rate ratios (RR) were calculated by event type (competition vs. practice) and division (U9/U11 vs. U13/U15). To account for clustering within league, generalized estimating equations with an exchangeable covariance structure were used for all analyses. Clusters (league) were based upon the level at which each athletic trainer covered youth lacrosse. RRs with 95 % confidence intervals (CIs) not including 1.00 were considered statistically significant.

## Results

### Overall frequencies and rates

During the 2015 season, 155 injuries were reported across 11,946AE, for an overall injury rate of 12.98/1000AE (95 % CI:10.93-15.02; Table 1). Most injuries occurred during games (60.0 %), were NTL (83.9 %). The TL-injury rate was 2.09/1000AE (95 % CI:1.27-2.891).

**Table 1:** Injury counts and rates in boys’ youth lacrosse, overall and by division, 2015 season

Division and event type	AEs^a^	Injury counts			Injury rates per 1000AE (95 % CI)		
		All injuries	Time loss injuries only^b^	Concussions	All injuries	Time loss injuries only	Concussion
Overall							
Game	4075	93	12	7	22.82 (18.18-27.46)	2.94 (1.28-4.61)	1.72 (0.45-2.99)
Practice	7871	62	12	3	7.88 (5.92-9.84)	1.65 (0.75-2.55)	0.38 (0.00-0.81)
Overall	11946	155	23	10	12.98 (10.93-15.02)	2.09 (1.27-2.91)	0.84 (0.32-1.36)
U9/U11							
Game	1151	31	1	1	26.93 (17.45, 36.41)	0.87 (0.00, 2.57)	0.87 (0.00, 2.57)
Practice	2029	17	2	2	8.38 (4.40, 12.36)	0.99 (0.00, 2.35)	0.99 (0.00, 2.35)
Overall	3180	48	3	3	15.09 (10.82, 19.36)	0.94 (0.00, 2.01)	0.94 (0.00, 2.01)
U13/U15							
Game	2924	62	11	6	21.20 (15.93, 26.48)	3.76 (1.54, 5.99)	2.05 (0.41, 3.69)
Practice	5842	45	11	1	7.70 (5.45, 9.95)	1.88 (0.77, 3.00)	0.17 (0.00, 0.51)
Overall	8766	107	22	7	12.21 (9.89, 14.52)	2.51 (1.46, 3.56)	0.80 (0.21, 1.39)

*NOTE*: *AE* athlete-exposure; *CI* confidence interval

^a^One athlete’s participation in one game/practice

^b^Injuries resulting in participation restriction of at least 24 h

Most injuries were to the lower extremity (45.2 %), and were diagnosed as contusions (51.6 %) and sprains (14.8 %; Table 2). Most injuries were due to equipment contact, particularly stick contact (35.5 %) and ball contact (14.2 %), followed by player contact (18.1 %; Table 3). In addition, common injury activities included general play (20.0 %), running (15.5 %), defending (12.9 %), and chasing a loose ball (12.3 %). Only three injuries (1.9 %) were reported due to checking.

**Table 2:** Injury counts, by body part and diagnosis, in youth boys’ lacrosse, overall and by division, 2015 season

	Overall		U9/U11		U13/U15	
	n	%	n	%	n	%
Body part						
Head/face	15	9.7	5	10.4	10	9.3
Neck	13	8.4	4	8.3	9	8.4
Shoulder	2	1.3	1	2.1	1	0.9
Arm/elbow	14	9.0	6	12.5	8	7.5
Hand/wrist	11	7.1	1	2.1	10	9.3
Trunk	22	14.2	9	18.8	13	12.1
Hip/groin	13	8.4	3	6.3	10	9.3
Thigh/upper leg	5	3.2	3	6.3	2	1.9
Knee	19	12.3	6	12.5	13	12.1
Lower leg	14	9.0	4	8.3	10	9.3
Ankle	17	11.0	1	2.1	16	15.0
Foot	2	1.3	0	0.0	2	1.9
Other	8	5.2	5	10.4	3	2.8
Diagnosis						
Concussion	10	6.5	3	6.3	7	6.5
Contusion	80	51.6	25	52.1	55	51.4
Inflammatory conditions	6	3.9	0	0.0	6	5.6
Spasm	7	4.5	4	8.3	3	2.8
Sprain	23	14.8	2	4.2	21	19.6
Strain	8	5.2	3	6.3	5	4.7
Other^a^	21	13.5	11	22.9	10	9.3
Total	155	100.0	48	100.0	107	100.0

^a^Includes abrasions (*n* = 2), environmental (*n* = 2), gastrointestinal (*n* = 2), knee pain (*n* = 2), respiratory (*n* = 2), and other injuries that were reported only once

**Table 3:** Injury counts, by injury mechanism and injury activity, in youth boys’ lacrosse, overall and by division, 2015 season

	Overall		U9/U11		U13/U15	
	n	%	n	%	n	%
Injury mechanism						
Player contact	28	18.1	9	18.8	19	17.8
Surface contact	12	7.7	5	10.4	7	6.5
Stick contact	55	35.5	20	41.7	35	32.7
Ball contact	22	14.2	4	8.3	18	16.8
Non-contact	19	12.3	1	2.1	18	16.8
Overuse	9	5.8	2	4.2	7	6.5
Illness/infection	5	3.2	5	10.4	0	0.0
Other/Unknown	5	3.2	2	4.2	3	2.8
Injury activity						
Blocking shot	5	3.2	1	2.1	4	3.7
Chasing loose ball	19	12.3	8	16.7	11	10.3
Checking	3	1.9	2	4.2	1	0.9
Conditioning	2	1.3	0	0.0	2	1.9
Defending	20	12.9	4	8.3	16	15.0
Face off	2	1.3	1	2.1	1	0.9
General play	31	20.0	10	20.8	21	19.6
Goaltending	6	3.9	2	4.2	4	3.7
Handling ball	2	1.3	0	0.0	2	1.9
Passing	8	5.2	3	6.3	5	4.7
Receiving Pass	4	2.6	1	2.1	3	2.8
Running	24	15.5	8	16.7	16	15.0
Shooting	17	11.0	4	8.3	13	12.1
Other	12	7.7	4	8.3	8	7.5
Total	155	100.0	48	100.0	107	100.0

Ten concussions (6.5 %) were reported. The ten concussions were due to player contact (*n* = 6), stick contact (*n* = 3), and ball contact (*n* = 1). The common injury activity was chasing a loose ball (*n* = 3). No anterior cruciate ligament (ACL) injuries were reported.

**Fig. 1:**
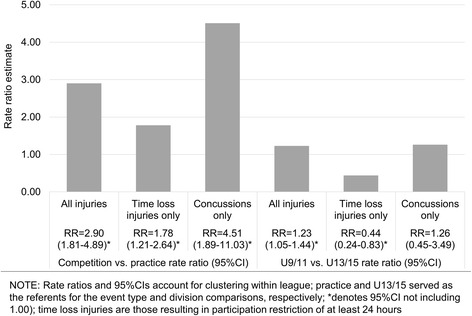
Rate ratios in boys’ youth lacrosse, by event type and division, 2015 season

The overall-injury rate was higher in games than in practices (RR = 2.90; 95 % CI:1.81-4.89; Fig. 1). This difference was attenuated when restricted to TL injuries only (RR = 1.78; 95 % CI:1.21-2.64), but higher when restricted to concussions only (RR = 4.51; 95 % CI:1.89-11.03).

### Comparisons by division

The U13/U15 divisions accounted for the largest proportion of overall injuries (69.0 %), TL injuries (92.0 %), and concussions (70.0 %; Table 1). All the TL injuries reported in the U9/U11 divisions were concussions. The overall-injury rate was higher in U9/U11 than U13/U15 division (RR = 1.23; 95 % CI:1.05-1.44; Fig. 1), but lower when restricted to TL injuries only (RR = 0.44; 95 % CI:0.24-0.83). Concussion rates did not differ between the U9/U11 and U13/15 divisions (RR = 1.26; 95 % CI:0.45-3.49).

Few differences were found when comparing the distribution of injuries in the U9/U11 and U13/U15 divisions (Table 2). Compared to the U9/U11 divisions, the U13/U15 divisions had larger proportions of injuries diagnosed as sprains (19.6 % vs. 4.2 %) and due to non-contact (16.8 % vs. 2.1 %). The U13/U15 divisions also had larger proportions of injuries due to ball contact (16.8 % vs. 2.1 %) and stick contact (16.8 % vs. 8.3 %).

## Discussion

Youth lacrosse is one of the fastest growing sports in the US (US Lacrosse “Participation…” 2015) Research in youth lacrosse is limited, yet necessary, to drive the development of interventions to reduce injury incidence and severity. Our study utilizes a large sample of boys’ youth lacrosse players across eight leagues in four states, and estimated an injury rate higher than that previously reported (12.98 vs. 8.7/1000AE) (Lincoln et al. 2014). However, our study included an additional younger division (U9). Lincoln et al. (2014) noted that the highest injury rate was found in the younger division (U11). In our study, the overall-injury rate in the U9/U11 divisions was higher than that of the U13/U15 divisions (15.09 vs. 12.21/1000AE). In contrast, the TL-injury rate in the U13/U15 divisions was higher than that of the U9/U11 divisions (2.51 vs. 0.94/1000AE). This is expected since upper youth divisions, according to US Lacrosse rules, allow for more bodily contact (US Lacrosse “^2015^ rules…” 2015).

TL-injury rates (game = 2.94/1000AE; practice = 1.65/1000AE) utilized an injury definition consistent with previous research (Dick et al. 2007; Xiang et al. 2014), and were found to be lower than those in college (game = 12.58/1000AE; practice = 3.24/1000AE) (Dick et al. 2007). Youth injury rates were similar to high school rates (game = 3.61/1000AE; practice = 1.51/1000AE) (Xiang et al. 2014), particularly within the U13/U15 divisions. The similar findings may be due to US Lacrosse rules allowing more bodily contact in upper divisions (US Lacrosse “^2015^ rules…” 2015). However, unlike high school and college, where most injuries were due to player contact and non-contact (Dick et al. 2007; Xiang et al. 2014), the largest proportion of youth lacrosse injuries were due to equipment contact, with over a third from stick contact. Potential injury mechanism differences may highlight the overall lower skill level of youth lacrosse compared with high school and college levels. Given our data originating from only one season, continued surveillance is required to obtain more precise estimates of potential variations by division within youth lacrosse and across competition levels.

The proportion of boys’ youth lacrosse injuries due to checking were low. The 2015 US Lacrosse Boys Youth Rules prohibit body checking in the U9/U11 divisions, and allow for limited body checking in the U13/U15 divisions (US Lacrosse “^2015^ rules…” 2015). Further efforts to ensure proper development of stick and body checking skills through coaching education and rules enforcement are warranted in throughout all divisions.

As in previous youth lacrosse research (Lincoln et al. 2014), most injuries were minor and diagnosed as contusions. Findings may vary from those at the high school and college levels (Dick et al. 2007; Xiang et al. 2014), where most injuries were sprains and concussions, due to our injury definition including NTL injuries (Kerr et al. 2015). Nevertheless, ten concussions were reported, with most occurring in competitions and from player contact. This is similar to recent research that analyzed video footage in high school boys’ lacrosse, finding that all 34 concussions captured were due to player contact (Lincoln et al. 2013). More definitive information on player and opponent activity would be useful to determine whether concussed players (or those that experience another injury) were defenseless. Previous research identified nearly half of all concussions in high school lacrosse to be associated with defenseless hits (Lincoln et al., 2013). If the trend holds at the youth level, efforts to enforce existing rules to limit defenseless hits may be warranted.

Although our sample originates from eight leagues in four states, findings were based on a small proportion of boys’ youth lacrosse players estimated in the United States and one season (US Lacrosse “Participation…” 2015). Our findings may not be generalizable to other youth lacrosse players. Under-diagnosis and/or underreporting of injuries may have occurred if youth players opted not to seek on-site care, or experienced delayed onset of symptoms after leaving the youth lacrosse setting. However, ATs are experienced professionals trained to accurately detect injury. Lastly, team- and league-based variations, such as coaching experience and certification (US Lacrosse “Certification…” 2015), the rate of participation growth (US Lacrosse “Participation…” 2015), skill level, and the numbers of games and practices across a season were not accounted for in the study, yet should be in future research.

Our boys’ youth lacrosse injury rate was higher than those previously reported. However, this estimate may be more precise given the larger sample. Continued surveillance across multiple seasons while accounting for coach-, team-, league-, and location-based variations will provide additional information regarding the epidemiology of youth lacrosse injuries. In addition, interventions may be warranted to reduce injury incidence. This can include adoption of age-appropriate rules to reduce exposure to player contact, enforcement of rules limiting defenseless hits, and ensuring proper development of checking skills through coaching education.

## Abbreviations

AT: Athletic trainer
NTL: Non-time loss
TL: Time loss
